# Modafinil In Debilitating fatigue After Stroke (MIDAS): study protocol for a randomised, double-blinded, placebo-controlled, crossover trial

**DOI:** 10.1186/s13063-016-1537-4

**Published:** 2016-08-17

**Authors:** Thomas Lillicrap, Venkatesh Krishnamurthy, John Attia, Michael Nilsson, Christopher R. Levi, Mark W. Parsons, Andrew Bivard

**Affiliations:** 1 Departments of Neurology, John Hunter Hospital, University of Newcastle, Newcastle, NSW Australia; 2 Hunter Medical Research Institute, University of Newcastle, Newcastle, NSW Australia

**Keywords:** Fatigue, Ischaemic stroke, Stroke recovery

## Abstract

**Background:**

Fatigue is a common symptom in stroke survivors for which there is currently no proven therapy. Modafinil is a wakefulness-promoting agent with established benefits in other disease models. We aim to test if modafinil will improve patient’s self-reported fatigue scores when compared to placebo and if therapy results in increased quality of life.

**Methods/design:**

MIDAS is a phase II, single-centre, prospective, double-blinded, randomised, crossover trial of modafinil for the treatment of persistent fatigue in survivors of ischaemic stroke. The inclusion criteria will require an average score of 12 or more across all domains of the Multi-dimensional Fatigue Inventory (MFI-20) and the diagnosis of a stroke more than 6 months prior. Patients will be randomised 1:1 to receive either modafinil 200 mg daily or placebo for a period of 6 weeks, after which a crossover will occur where patients who are on modafinil will begin taking placebo and vice versa. The primary outcome will be improvement in fatigue as measured by the MFI-20. Secondary outcomes will include changes in the Fatigue Severity Scale, improved cognition measured using the Montreal Cognitive Assessment, improvement in mood as determined by the Depression, Anxiety and Stress Scale and improvement in each patient’s stroke-specific quality of life score. All participants will also undergo magnetic resonance imaging (MRI) at baseline, crossover and study conclusion to measure cerebral blood flow on arterial spin labelling and brain activity on resting state functional MRI. This study will comply with the CONSORT guidelines. The projected sample size requirement is 36 participants in a crossover trial giving a power of 80 % and a type-1 error rate of 0.05.

**Discussion:**

MIDAS seeks to enhance the quality of life in stroke survivors by assisting or resolving stroke-associated fatigue.

**Trial registration:**

ACTRN12615000350527, registered on the 17 April 2015.

Protocol version 3, approved 16 June 2015.

**Electronic supplementary material:**

The online version of this article (doi:10.1186/s13063-016-1537-4) contains supplementary material, which is available to authorized users.

## Background

Severe fatigue is reported in approximately half of all stroke survivors [1, 2] and can persist for years after the index event, resulting in a reduction in quality of life. Fatigue is a common symptom in chronic disease and is hypothesised to be caused by a combination of organic brain lesions and psychosocial stress related to lifestyle adjustment. Fatigue can also be caused by sleep apnoea [3] and is a pivotal component of depression [4], which is also common after stroke. Fatigue after stroke has been identified to be an independent predictor of poor outcome [5]; whether this is due to the severity of the stroke itself or to fatigue directly contributing to poor outcome is not yet understood. However, neuroimaging studies have found no clear association between characteristics of the brain lesion (e.g. location, pathological type) and fatigue [6]. Currently there are no proven therapies for post-stroke fatigue. In the present study, we seek to investigate if 200 mg daily of modafinil can improve post-stroke fatigue and other aspects of quality of life in a community-dwelling population.

Modafinil is a nonamphetamine wakefulness-promoting agent with limited side effects that is currently used to treat excessive sleepiness associated with narcolepsy, shift-work sleep disorder and obstructive sleep apnoea. Modafinil has also been used in the treatment of fatigue associated with Parkinson’s disease, multiple sclerosis, traumatic brain injury and post-polio syndrome [7]. Several case reports advocating the use of modafinil in stroke patients have been published [8, 9]. However, a recent study of modafinil treatment immediately after stroke was negative due to resolution of fatigue in both the control and therapy groups [10]. We hypothesised that treatment with modafinil would reduce the severity of self-reported fatigue in stroke survivors compared to placebo.

## Methods/design

Modafinil In Debilitating fatigue After Stroke (MIDAS) will be a phase II, single-centre, prospective, double-blinded, randomised, crossover trial to investigate if modafinil reduces the burden of fatigue after ischaemia in patients who report ongoing severe fatigue (as measured by the Multidimensional Fatigue Inventory (MFI-20)). This study will apply the Consolidated Standards of Reporting Trials (CONSORT) and the Standard Protocol Items: Recommendations for Interventional Trials (SPIRIT) (Additional file 1: Figure S1) [11] guidelines. The project protocol was developed by researchers from the University of Newcastle without external influence. Data management includes assigning participants unique study identifiers and scanning participant documents and backup on secured computers as detailed in the protocol. The projected total sample size requirement is 36 participants. Ethical approval was granted by the Hunter New England Human Research Ethics Committee in accordance with the Helsinki Deceleration on 9 June 2015, reference 15/04/15/3.02. Because this is a single-centre study of 36 participants, no Data Safety Monitoring Board will be required.

### Patient population

Patients will be recruited following from the Newcastle, NSW, Australia community and from stroke clinics at John Hunter Hospital. Inclusion criteria will be patients aged over 18 years with a history of stroke at least 6 months previously and an average score of 12 across each domain of the MFI-20. All participants are required to provide informed consent.

Inclusion criteria:

- Being 18 years of age or older
- Having suffered a stroke or transient ischaemic attack (TIA) at least 3 months previous
- Having persistent self-reported fatigue with a MFI-20 score of 12 or more
- A modified Rankin Score (mRS) of 3 or less
- Able to speak reasonable English, understand instructions and be able to complete tests and questionnaires on their own or with minimal support
- Able to give informed consent

Exclusion criteria:

- Having pre-existing depression, dementia or other neuropsychiatric disease
- Having other diagnoses with fatigue as a known symptom, e.g. chronic fatigue syndrome, multiple sclerosis
- Having stroke induced by trauma, infection or surgery
- Current or past drug abuse
- Known contraindication to treatment with modafinil
- Known active malignancy, any intracranial tumor, subdural or epidural hematoma
- Known contraindications to MRI scanning, e.g. claustrophobia, cardiac pacemaker or other implants
- Known cause of fatigue (i.e. sleep apnoea)
- Having renal or hepatic impairment
- Use of benzodiazepines or antiepileptic drugs
- Patients on immunosuppression or known immunodeficiency state, e.g. HIV

Study drug will be discontinued if there are any adverse events due to the drug (i.e. rash).

### Therapy

Participants will be randomised 1:1 to modafinil 200 mg per day or placebo. All participants will be provided with 6 weeks’ worth of study drug after randomisation. After 6 weeks, participants will be asked to return to the study centre for assessments and to return study drug bottles to assess compliance. After the study drug has been returned, participants will have a 1-week washout period. Once the washout period has been completed, participants will again be asked to return to the study centre and crossover will occur where participants who initially received modafinil will be given a placebo or those who were given placebo will receive modafinil. Lastly, after 6 weeks on the second round of the study drug, participants will be asked to return to the study centre for assessments and to return study drug bottles to assess compliance.

### Assessments

**Fig. 1:**
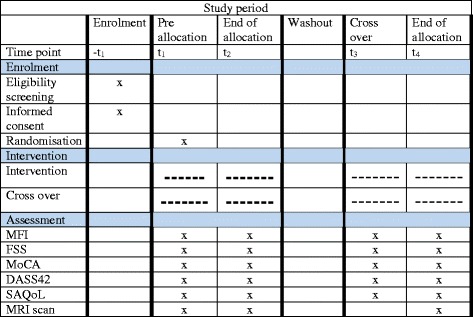
Standard Protocol Items: Recommendations for Interventional Trials (SPIRIT) diagram for the MIDAS trial. A SPIRIT diagram of the study procedures. Following enrolment, patients are randomised to either active drug or placebo. Following 6 weeks of therapy and a 1-week washout, patients are crossed over either to active drug or placebo. All patient assessments are carried out at the start and conclusion of each therapy round. MRI scans are performed at the start of the study and at the end of each therapy round. *DASS 42,* Depression, Anxiety and Stress Scale, *FSS,* Fatigue Severity Scale, *MFI,* Multidimensional Fatigue Inventory, *MoCA,* Montreal Cognitive Assessment, *MRI* magnetic resonance imaging, *SSQoL,* Stroke-associated Quality of Life

Assessments will be carried out at baseline, in the last week of the first 6-week treatment arm, after a 1-week washout period and in the last week of the second 6-week treatment arm (Fig. 1). The assessment at each time point will include the MFI-20, the Montreal Cognitive Assessment (MoCA), the Fatigue Severity Scale (FSS), the Depression, Anxiety and Stress Scale (DASS 42) and the Stroke-specific Quality of Life (SSQoL) scale. All assessments will be carried out by research staff blinded to treatment. All participants will also complete magnetic resonance imaging (MRI) sessions at baseline, crossover and study conclusion to measure cerebral blood flow on arterial spin labelling (ASL) and brain activity on resting state functional MRI (rsfMRI). Drug adherence will be monitored through tablet return for each patient in each strata (placebo or active drug).

### Consent and randomisation

Participant consent will be obtained following screening with the MFI-20 by an independent researcher who is not involved with the routine care of the participant. Participants will be referred to the research team by clinicians who see stroke survivors in clinics. Where appropriate the clinician will introduce the study to potential participants if the clinician believes that the potential participant will meet the inclusion criteria and be willing and able to provide informed consent as well as maintain study enrolment for the full length of the trial.

Patients will be randomly allocated to taking either 200 mg modafinil or an identical placebo daily. Treatment allocation will be performed by the local pharmacy according to a randomisation schedule generated by an independent statistical support unit (Clinical Research Design, IT, and Statistical Support, CReDITSS) at the Hunter Medical Research Institute. Randomisation will be based on a computer-generated list of random numbers with varying block size, linked to a unique treatment number. All study participants and researchers outside of the pharmacy will be blinded to treatment allocation. Unblinding will occur in the event of an adverse or serious adverse event and will be performed by the pharmacy at the request of the study researchers or clinicians. A serious adverse events related to modafinil therapy can include developing a rash, or any event causing study participant withdrawal or hospitalisation while in the study. An adverse event will be defined as the occurrence of headache, nausea, anxiety/nervousness, insomnia, and diarrhoea.

### Primary outcomes

The primary outcome will be a significant reduction in the self-reported MFI-20 score during modafinil treatment compared to that participant’s difference during the placebo period.

### Secondary outcomes

Secondary analyses will assess the effect of treatment with 200 mg modafinil daily to baseline assessments of cognition (MoCA), mood (DASS 42) and quality of life (SSQoL). We will also seek a repeated measure of fatigue with the FSS which is used more widely yet is much less specific for the source of type of fatigue.

Secondary analysis will also explore the MRI changes associated with administering 200 mg modafinil daily in terms of cerebral blood flow on ASL and brain activity on rsfMRI.

### MRI analysis plan

Secondary analysis will be performed on unblinded patient data to compare with MRI findings. Resting state functional MRI (rsfMRI) data will be analysed using independent component analysis to determine whether there are different network activation patterns on active drug or placebo. Arterial spin labelling (ASL) will also be analysed to determine whether there are any changes in the whole brain cerebral blood flow between when participants are on active drug or placebo. Lastly, the SSQoL, MoCA, DASS 42 and MFI-20 will be analysed in conjunction with rsfMRI data to identify whether a change in any score is associated with alterations in resting state network function.

### Statistical analysis plan

A linear mixed model will be used to detect a significant difference between final MFI-20 scores during each period as the outcome. The value of the baseline MFI-20 score at trial start and after crossover will be used as a covariate, and a group variable as the exposure of interest in order to correct for changes in baseline MFI-20 scores; an order term will also be included to make sure that there is no carryover effect, i.e. no difference in effect if the active drug or placebo is given first.

Secondary outcomes will be assessed using the same methods as for the MFI analysis but using the MoCA, DASS42, SSQoL and FSS instead. MRI analysis will be undertaken using the same methods as for the primary analysis.

Given that all secondary outcomes are also continuous, the same approach will be used for analysis of these outcomes.

### Sample size

The study was designed to have 80 % power in detecting a 10 point decrease in self-reported fatigue on the MFI after 6 weeks of modafinil treatment with a type I error rate of 0.05 and assuming standard deviation in the patient population of 14. Using previously reported effects of modafinil on fatigue for stroke survivors [10], multiple sclerosis [12] and a Cochrane review [13] a total sample size of 34 is required for a crossover study. Assuming modest participant dropout and study drug compliance, we aim to recruit 36 participants in total. The crossover design reduces the number of participants required and the negative effects of missing data given the small number of dropouts expected [14].

## Discussion

MIDAS seeks to enhance the quality of life in stroke survivors by assisting or resolving stroke-associated fatigue. There are limited interventions available to stroke survivors with scant strong evidence establishing their efficacy. The proposed crossover trial is an ideal study design as the patient disability being examined is already established, meaning that patients can be enrolled for a longer period of time and the fatigue has not spontaneously resolved as has occurred in an earlier time frames post stroke [10]. Additionally, by setting the strict inclusion criterion of an average MFI-20 score of 12 across all domains, we are ensuring that patients have established severe fatigue and are able to rule out milder cases, which may not show a significant change in self-reported scores. Such individual patient selection has been the bedrock of recent stroke trials and is a key to effective therapy delivery. Fatigue is a major health concern for stroke survivors; it reduces their quality of life and limits engagement in rehabilitation activities. An effective therapy may have profound implications for stroke patients’ quality of life.

### Trial status

At the time of writing 24 of the proposed 36 patients have been enrolled.

## Additional file

Additional file 1: Figure S1. SPIRIT checklist for the MIDAS trial. SPIRIT 2013 checklist detailing the MIDAS trial-specific information as described in the protocol. (PDF 642 kb)

## Abbreviations

ASL: Arterial spin labelling
CONSORT: Consolidated standards of reporting trials
CReDITSS: Clinical research design, IT, and statistical support
FSS: Fatigue severity scale
MFI: Multi-dimensional fatigue inventory
MIDAS: Modafinil in debilitating fatigue after stroke
MoCA: Montreal cognitive assessment
MRI: Magnetic resonance imaging
rsfMRI: Resting state functional magnetic resonance imaging
SSQoL: Stroke-specific quality of life
TIA: Transient ischaemic attack
